# Kumujan B suppresses TNF-α-induced inflammatory response and alleviates experimental colitis in mice

**DOI:** 10.3389/fphar.2024.1427340

**Published:** 2024-08-01

**Authors:** Xunwei Li, Qianqian Di, Xiaoli Li, Xibao Zhao, Han Wu, Yue Xiao, Haimei Tang, Xucan Huang, Jin Chen, Shaoying Chen, Yuli Gao, Junbo Gao, Weilie Xiao, Weilin Chen

**Affiliations:** ^1^ School of Pharmaceutical Sciences, Marshall Laboratory of Biomedical Engineering, Shenzhen University, Shenzhen, China; ^2^ Guangdong Provincial Key Laboratory of Regional Immunity and Diseases, Department of Immunology, Institute of Biological Therapy, Shenzhen University Medical School, Shenzhen University, Shenzhen, China; ^3^ Key Laboratory of Medicinal Chemistry for Natural Resource, Ministry of Education, Yunnan Characteristic Plant Extraction Laboratory, Yunnan Provincial Center for Research and Development of Natural Products, School of Pharmacy and School of Chemical Science and Technology, Yunnan University, Kunming, China; ^4^ Southwest United Graduate School, Kunming, China

**Keywords:** Kumujan B, TNF-α, inflammatory bowel disease, inflammation, β-carboline alkaloids

## Abstract

Treatments of inflammatory bowel disease (IBD) are diverse, but their efficacy is limited, and it is therefore urgent to find better therapies. Controlling mucosal inflammation is a must in IBD drug treatment. The occurrence of anti-tumor necrosis factor α (TNF-α) monoclonal antibodies has provided a safer and more efficacious therapy. However, this kind of treatment still faces failure in the form of loss of response. β-Carboline alkaloids own an anti-inflammatory pharmacological activity. While Kumujan B contains β-carboline, its biological activity remains unknown. In this study, we attempted to determine the anti-inflammatory effects of Kumujan B using both the TNF-α- induced in vitro inflammation and DSS-induced in vivo murine IBD models. Our data show that Kumujan B attenuated the expression of interleukin 1β (IL-1β) and interleukin 6 (IL-6) induced by TNF-α in mouse peritoneal macrophages. Kumujan B suppressed c-Jun N-terminal protein kinases (JNK) signaling, especially c-Jun, for anti-inflammatory response. Furthermore, Kumujan B promoted K11-linked ubiquitination and degradation of c-Jun through the proteasome pathway. In an *in vivo* study, Kumujan B inhibited the expression of IL-1β, IL-6, and TNF-α and improved the colon barrier function in dextran sulfate sodium salt (DSS)-induced experimental mice colitis. Kumujan B exhibited *in vivo* and *in vitro* anti-inflammatory effects, making it a potential therapeutic candidate for treating IBD.

## Graphical Abstract

**Figure:**
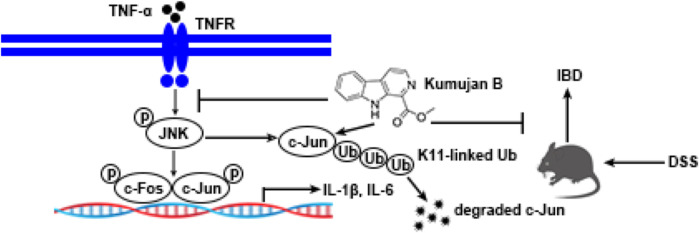

## 1 Introduction

Inflammatory bowel disease (IBD) mainly comprises Crohn’s disease (CD) and ulcerative colitis (UC), which are two distinct but related diseases (Retnakumar and Muller, 2019). In the Western world, such as northern Europe and North America, the incidence rate of IBD is the highest worldwide. However, the prevalence of IBD is increasing in Asia, an area with a previously low incidence of IBD (Baumgart and Carding, 2007). IBD is spreading rapidly in China, much like it did in Western countries (Kaplan and Ng, 2016). The pathogenesis of IBD is still not fully understood, but environmental and genetic factors play an important role. Patients with IBD have the following typical symptoms: chronic diarrhea, abdominal pain, rectal bleeding, and fatigue (Neurath, 2019; Agrawal et al., 2022). The treatment of IBD is diverse, including 5-aminosalicylic acid (5-ASA), corticosteroids, immunomodulators, and anti-tumor necrosis factor α (TNF-α) drugs. Their limited efficacy, such as loss of response and/or adverse effects, cannot be ignored. Therefore, it is urgent to discover new therapeutic drugs with fewer side effects (Bernstein, 2015; Wright et al., 2018; Agrawal et al., 2021).

Major inflammatory mediator TNF-α is primarily produced through activated macrophages (Beutler and Cerami, 1989; Masola et al., 2022). TNF-α exerts its biological effects by two distinct receptors, TNF receptor 1 (TNFR1) and TNF receptor 2 (TNFR2). TNFR1 is expressed universally, while TNFR2 is only expressed in specific cell types, such as neurons, immune cells, and endothelial cells. TNF-α produced by stimulated macrophages can bind to either TNFR1 or TNFR2 directly (Paul et al., 2006; Kalliolias and Ivashkiv, 2016), whereas TNFR1 initiates most of TNF’s biological activities. TNF-α binds to TNFR1, inducing receptor trimerization and recruitment of the adapter protein TNF receptor-associated death domain (TRADD). TRADD recruits TNF receptor-associated factor 2 (TRAF2), which then recruits and activates the IKK complex (IKKα, IKKβ, and NEMO) by the receptor-interacting protein (RIP). Ubiquitinated RIP1 activates TAK1, and in sequence, TAK1 activates IKKα and IKKβ. IKKβ is crucial for the phosphorylation, ubiquitination, and degradation of the inhibitor of NF-κB (IκBα). The degradation of IκBα results in translocation of NF-κB to the nucleus, which stimulates the transcription of pro-inflammatory cytokines and chemokines (Wu and Zhou, 2010; Chu, 2013).

TRAF2 also activates the mitogen-activated protein kinase kinase kinases (MAPKKK, also known as MEKK). The phosphorylation of MAPKKK activates downstream protein kinases MAPK kinases (MAPKK, also known as MEK). In sequence, MAPKK phosphorylates the mitogen-activated protein kinases (MAPK). MAPK includes three subfamilies: c-Jun N-terminal protein kinases (JNK)/stress-activate protein kinases (SAPK), p38 MAPK, and extracellular regulated protein kinases (ERK) in mammals. The activation of the JNK MAPK pathway can phosphorylate downstream transcription factors Fos (c-Fos, FosB, Fra-1, and Fra-2) and Jun (c-Jun, JunB, and JunD), increasing the formation of activator protein 1 (AP-1) complexes and altering the response to cytokines. Fos proteins can only heterodimerize with the Jun family, while Jun proteins can both homodimerize and heterodimerize with Jun or Fos proteins. Among these complexes, c-Jun acts as a major member (Chen and Goeddel, 2002; Raivich et al., 2004; Cui et al., 2007; Ye et al., 2014). TNFR2 binds preferentially with TNF-α and combines directly with TRAF2 (Mancusi et al., 2019). TNFR2 can also activate NF-κB and MAPK signaling, resulting in a series of changes in inflammation, cell proliferation, and cell survival (Borghi et al., 2016; Siegmund et al., 2022).

Traditional medicine can deal with most basic healthcare needs, according to the World Health Organization (WHO). This kind of therapy mainly requires herbal extracts and their active components. These substances have shown their pharmacological properties against diseases (Arulselvan et al., 2016). β-Carbolines are a class of indole alkaloids containing a tricyclic pyrido [3,4-b] indole ring system. They are isolated from the plant species *Peganum harmala* and have multiple pharmacological activities (Aaghaz et al., 2021; Luo and Song, 2021), such as neuropharmacological effects, anti-tumor effects, anti-oxidant effects, anti-inflammatory effects, anti-microbial, and anti-viral effects (Xie et al., 2021). Kumujan B, found in *Lycium chinense* and *Alstonia constricta*, belongs to the β-carboline group. Its molecular formula is C_13_H_10_N_2_O_2_, and it has a relative molecular mass of 226.23. However, the anti-inflammation effect of Kumujan B has not been reported. In this study, we found that Kumujan B could significantly suppress the expression of pro-inflammatory cytokines IL-1β and IL-6 by downregulating the JNK MAPK signaling pathway in TNF-α-induced inflammation. Furthermore, Kumujan B could interact with c-Jun and promote its degradation via K11-linked ubiquitination. An *in vivo* study showed that Kumujan B alleviated DSS-induced experimental colitis in mice. Together, this may provide a potential therapeutic agent for IBD.

## 2 Materials and methods

### 2.1 Reagents and antibodies

Methyl sulfoxide (DMSO, Lot: D5879) was purchased from Sigma (United States), cycloheximide (CHX, Lot: S7418), Z-Leu-D-Leu-Leu-al (MG132, Lot: S2619), and chloroquine (CQ, Lot: S6999) were purchased from Selleck (United States). TNF-α (Cat: 315-01A) was obtained from PeproTech (United States). Dextran sulfate sodium salt (DSS) was purchased from MP Biomedicals (CA, United States). The antibodies against E-cadherin, IKKα, IKKβ, p-IKKα/β, IκBα, p-IκBα, p65, p-p65, JNK, p-JNK, ERK, p-ERK, p38, p-p38, p-c-Fos, p-c-Jun, and c-Jun were obtained from Cell Signaling Technology (United States). Antibodies specific to β-actin, GAPDH, and Myc were purchased from Proteintech (United States). Myc-c-Jun plasmid was purchased from Hanyi (Guangzhou, China), and the HA-Ub, HA-K6, HA-K11, HA-K27, HA-K29, and HA-K33 plasmids were purchased from Addgene (United States).

### 2.2 Kumujan B

We screened an in-house library of natural products. Kumujan B (CAS: 3464-66-2) showed bioactivity in anti-inflammation. The compound we used in this study was isolated and purified at Yunnan University, and the isolation processes are as follows. The seeds of *Nigella glandulifera* were extracted with 70% acetone–water at room temperature and filtered. The extract was subjected to a silica gel column and eluted with a CHCl_3_−MeOH gradient system to afford fractions A−E. Fraction A was further purified by RP-18 column chromatography (MeOH−H_2_O gradient, 60%–100%), followed by semi-preparative HPLC (86% MeOH−H_2_O) to obtain compound Kumujan B.

### 2.3 Mice and cell culture

Wild-type C57BL/6 mice (female, 8 weeks, 17–20 g) were purchased from Guangdong Medical Laboratory Animal Center (Guangdong, China). The mice were housed in individual and specific pathogen-free conditions. Mice were maintained in cages (four mice per cage) with a 12-h light/dark cycle. All animal experiments carried out according to protocols were permitted by Shenzhen University Medical School (Approval Number: A202301428) and complied with the Guidelines on Animal Welfare of Shenzhen University Medical School.

Mouse peritoneal macrophages were isolated from C57BL/6 mice after intraperitoneal injection of 2 ml of 3% thioglycolate broth for 4 days. The mouse peritoneal macrophages were cultured in RPMI 1640 with 10% FBS and antibiotics. HEK293T cells were cultured in DMEM to which was added 10% FBS and antibiotics.

### 2.4 Animal experiments

For DSS-induced mouse colitis, mice were randomly divided into four groups: a DMSO + water group, a Kumujan B (10 mg/kg) + water group, a DMSO +3.5% DSS group, and a Kumujan B (10 mg/kg) + 3.5% DSS group (n = 4 per group). DMSO or Kumujan B (10 mg/kg) treatment was performed by gavage daily. After 7 days, the drinking water of the DSS groups was changed to distilled water for 1 day. All mice were sacrificed on the eighth day. Colon tissues were collected for subsequent study.

### 2.5 Histological analysis

Tissues of the heart, liver, spleen, lung, kidney, and colon tissues of the mice were fixed in 4% paraformaldehyde and then embedded in paraffin, sectioned, and stained with hematoxylin and eosin (H&E). All colon tissue sections were scored according to a previously published grading system (Adolph et al., 2013). This grading system consists of five histological subscores. For each parameter: 0, absent; 1, mild; 2, moderate; 3, severe: mononuclear cell infiltrate (0–3), crypt hyperplasia (0–3), epithelial injury/erosion (0–3), polymorphonuclear cell infiltrates (0–3), and transmural inflammation (0–4). The percentage of the area involved in the inflammation process was the basis of the extent factor: 1, <10%; 2, 10%–25%; 3, 25%–50%; and 4, >50%.

### 2.6 Evaluation of disease activity index (DAI)

To assess the severity of colitis, we combined body weight loss, stool consistency, and rectal bleeding as DAI scores. The criteria we used were published in a previous article, and each sign consists of five subscores (Wirtz et al., 2017). In brief, body weight loss: none, score 0; 1%–5%, score 1; 6%–10%, score 2; 11%–18%, score 3; >18%, score 4. Stool consistency: normal, score 0; soft but still formed, score 1; soft, score 2; very soft, wet, score 3; watery diarrhea, score 4. Blood: negative hemoccult (no bleeding), score 0; negative hemoccult, score 1; positive hemoccult, score 2; blood traces in stool visible, score 3; gross rectal bleeding, score 4.

### 2.7 Cell viability assay

The cell viability was detected by CCK8 and flow cytometry assays. Mouse peritoneal macrophages were plated in 96-well plates (10^4^ cells/100 μL/well) and cultured in 5% CO_2_ at 37°C overnight. The cells were exposed to DMSO or different concentrations of Kumujan B (0 μM, 12.5 μM, 25 μM, or 50 μM) for 12 h. Then, the culture medium was exchanged for fresh medium with CCK8, and the plates were incubated at 37°C for 2 h according to the manufacturer’s instructions (Yeasen, Shanghai, China). The absorbance value of each well was read at 450 nm (A450) by using a microplate reader.

Mouse peritoneal macrophages were collected and plated in 12-well plates (10^6^ cells per well) and cultured in 5% CO_2_ at 37°C overnight. Thereafter, the cells were treated with DMSO or different concentrations of Kumujan B (0 μM, 12.5 μM, 25 μM, or 50 μM) for 12 h and then stained with Annexin V-FITC (Yeasen, Shanghai, China) and PI (Yeasen, Shanghai, China) in binding buffer. The samples were detected by flow cytometry (CytoFLEX, Beckman Coulter, United States). Isotype control antibodies were used as control, and annexin V^+^ PI^+^ was used to detect dead cells.

### 2.8 Western blotting

Mouse peritoneal macrophages were pretreated with DMSO or Kumujan B for 3 h and then incubated with TNF-α (100 ng/ml) for the indicated time. Supernatants were collected for further study, and cells were washed with PBS twice. Cells were lysed by 1x NP40 lysis buffer containing a 1x protease inhibitor mixture and centrifuged for 5 min at 12,000 g. RIPA lysis buffer containing a 1x protease inhibitor mixture was used to isolate proteins from colon tissues. After being ground into small pieces, samples were centrifuged for 5 min at 12,000 g. The cell or tissue lysates were used to determine the protein concentrations using BCA protein assay kit (Beyotime Biotechnology, China) according to the manufacturer’s instructions. Equal amounts of protein were subjected to 10% SDS-PAGE gels, transferred onto NC membranes, blocked with 5% skim milk, and then incubated with the membranes with the corresponding primary antibodies at 4°C overnight. On the next day, the samples were rinsed with TBST three times, followed by the incubation of secondary antibodies for 2 h at room temperature. In order to visualize the protein bands, we used FluorChem E (Cell Biosciences, Santa Clara, United States) to record the signals.

### 2.9 Ubiquitination assay

HEK293T cells were transfected with Myc-c-Jun, HA-Ub, HA-K6, HA-K11, HA-K27, HA-K29, and HA-K33 plasmids with PEI reagents for 24 h following the manufacturer’s instructions. Cells were pretreated with MG132 (40 μM) for 1 h and then treated with DMSO or Kumujan B (25 μM) for 4 h. Cells were collected and lysed with lysis buffer containing protease inhibitor cocktail (Bimake, United States) and 1% SDS. The cell lysate was collected, and a quarter of it was used for the whole cell lysate (WCL). The remaining lysate was immunoprecipitated with anti-Myc antibody for 2 h, and then protein A/G magnetic beads (Selleck, United States) were added to the lysate overnight at 4°C. The beads were then washed, and the proteins of interest were released by adding SDS sample buffer to the lysate. All samples were detected by Western blotting.

### 2.10 Quantitative PCR

Total RNA was extracted from mouse peritoneal macrophages or colon tissues using TRIzol regent (Takara, Japan) and was reverse-transcribed into cDNA according to the manufacturer’s instructions (Takara, Japan). cDNA was subjected to Q-PCR analysis. This analysis used Hieff^®^ qPCR SYBR®Green Master Mix (Yeasen, Shanghai, China) and a CFX96 Touch Real-Time PCR System (Bio-Rad, Hercules, CA, United States), according to the manufacturer’s instructions. Data were normalized for the expression of β-actin in each sample. Specific primers used for the Q-PCR assay are as follows: mIL-1β (forward, 5′-AAC​TGT​TCC​TGA​ACT​CAA​CTG​T-3′; reverse, 5′-GAG​ATT​TGA​AGC​TGG​ATG​CTC​T-3′), mTNF-α (forward, 5′-AAG​CCT​GTA​GCC​CAC​GTC​GTA-3′; reverse, 5′-GGC​ACC​ACT​AGT​TGG​TTG​TCT​TT-3′), and mβ-actin (forward, 5′-AGT​GTG​ACG​TTG​ACA​TCC​GT-3′; reverse, 5′-GCA​GCT​CAG​TAA​CAG​TCC​GC-3′).

### 2.11 ELISA

The protein levels of mouse IL-1β, IL-6, and TNF-α were measured by ELISA kits (eBioscience Biotechnology, CA, US). The expressions of glutamic oxaloacetic transaminase (AST) and creatine kinase (CK) in serum were detected by ELISA kits (BY abscience, Jiangsu, China). Detection of the above-mentioned indexes was done according to the manufacturer’s instructions.

### 2.12 Creatinine (CR) detection

The expression of CR in mice serum was detected by a CR LiquiColor kit (Stanbio Laboratory, TX, US).

### 2.13 Molecular docking study

The 3D structure of Kumujan B was downloaded from the PubChem database. The source of the c-Jun protein structure was the AlphaFold Protein Structure Database with the identifier AF-P05412-F1. A molecular docking study was performed using Autodock version 4.2.6. Water was removed, and hydrogens were added as preparation. The default parameters were set. The best computational interaction mode of Kumujan B and c-Jun protein was visualized using a drawing by PyMOL.

### 2.14 Statistical analysis

All data are presented as mean ± SD and collected from three independent sets of experiments. GraphPad Prism 6.0 was used for plotting data. Student’s t-test was used to determine the differences between the two groups. *p* values <0.05 were considered to be statistically significant.

## 3 Results

### 3.1 Kumujan B does not affect cell viability in mouse peritoneal macrophages

**FIGURE 1:**
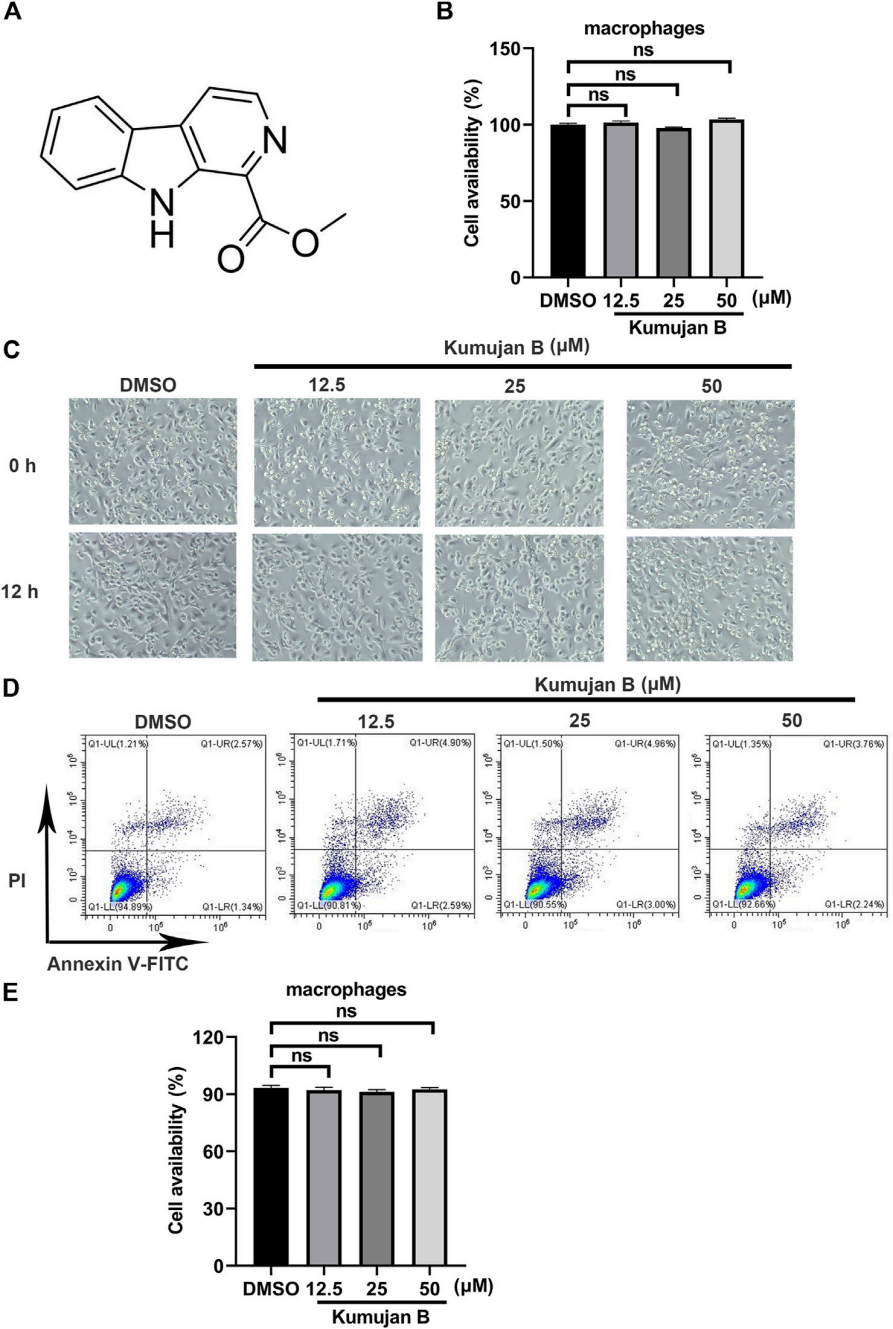
Kumujan B does not affect the cell viability of mouse peritoneal macrophages. **(A)** The chemical structure of Kumujan B. **(B)** Mouse peritoneal macrophages were plated in 96-well plates overnight and then treated with DMSO or different concentrations of Kumujan B (12.5 μM, 25 μM, and 50 μM) for 12 h. The cell viability was determined using a CCK8 assay. **(C)** The morphology of mouse peritoneal macrophages was observed under a microscope. **(D)** The cytotoxicity of Kumujan B in mouse peritoneal macrophages was detected by flow cytometry. **(E)** The statistical analysis result of flow cytometry. All results obtained from three independent experiments, and values are presented as the mean ± SD. ns, not significant.

Kumujan B is a compound isolated from the seeds of *Nigella glandulifera*, which belongs to the group of β-carboline. Its chemical structure is shown in Figure 1A. The cytotoxicity of Kumujan B in mouse peritoneal macrophages was tested with a CCK8 assay. The mouse peritoneal macrophages were incubated with Kumujan B at different concentrations (0 μM, 12.5 μM, 25 μM, and 50 μM) for 12 h, and the results demonstrated that Kumujan B did not affect the viability of mouse peritoneal macrophages (Figure 1B). The cell morphology was observed under the microscope. The generated images indicated that the treatment of Kumujan B up to 50 μM had no obvious cytotoxicity to mouse peritoneal macrophages (Figure 1C). A similar result was also shown by flow cytometry analysis. Mouse peritoneal macrophages maintain viability without cytotoxic effect when incubated with Kumujan B (0–50 μM) for 12 h (Figures 1D, E). Hence, in subsequent cellular experiments, concentrations of Kumujan B within the safe concentration range could be selected for use.

### 3.2 Kumujan B suppresses TNF-α-induced inflammatory response in mouse peritoneal macrophages

**FIGURE 2:**
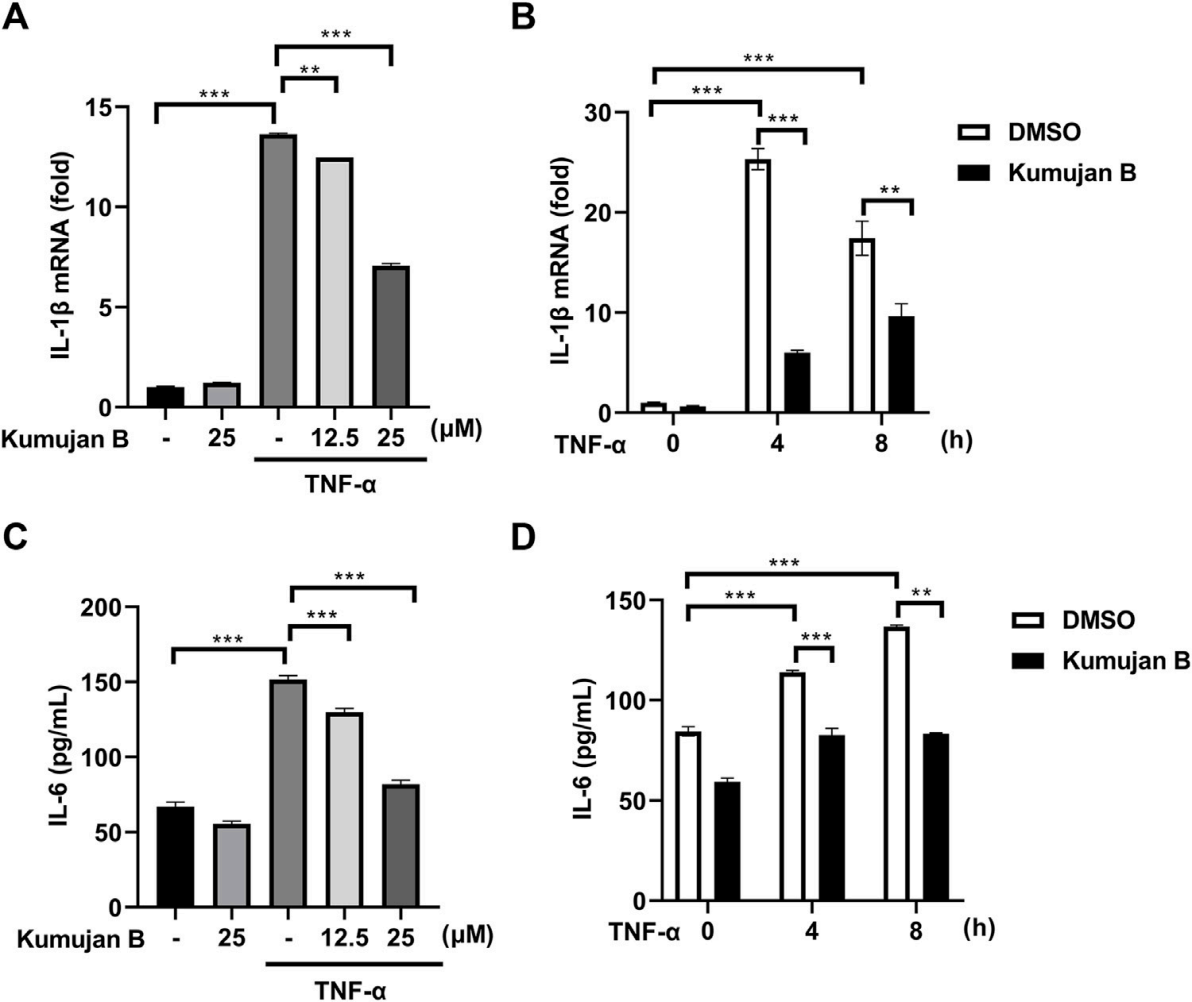
Kumujan B suppresses pro-inflammatory cytokine expression in TNF-α-stimulated mouse peritoneal macrophages. **(A)** Q-PCR analyzed the mRNA expression of IL-1β after mouse peritoneal macrophages were pretreated with DMSO or different concentrations of Kumujan B for 3 h and then stimulated by TNF-α (100 ng/mL) for 6 h. **(B)** Q-PCR analyzed the mRNA expression of IL-1β. Mouse peritoneal macrophages were pretreated with DMSO or Kumujan B (25 μM) for 3 h and then stimulated by TNF-α (100 ng/mL) for the indicated time. **(C)** The secretion of IL-6 in supernatants was detected by ELISA. Mouse peritoneal macrophages were pretreated with DMSO or Kumujan B (12.5 μM or 25 μM) for 3 h and then stimulated by TNF-α (100 ng/mL) for 8 h. **(D)** The secretion of IL-6 in supernatants was detected by ELISA. Mouse peritoneal macrophages were pretreated with DMSO or Kumujan B (25 μM) for 3 h and then stimulated by TNF-α (100 ng/mL) for the indicated time. All results obtained from three independent experiments, and values are presented as the mean ± SD. *p < 0.05, **p < 0.01, and ***p < 0.001.

To further confirm the effect of Kumujan B on TNF-α-induced inflammation, we chose macrophages and immune cells that contribute to the inflammatory response in colons (Pan et al., 2022) as test subjects of *in vitro* assays. We pretreated cells with DMSO or Kumujan B for 3 h and then stimulated with TNF-α (100 ng/ml) for 6 h. The expression of IL-1β was detected by Q-PCR. As shown in Figure 2A, Kumujan B suppressed the expression of IL-1β in a dose-dependent manner. Results also showed that Kumujan B (25 μM) repressed the expression of IL-1β in mouse peritoneal macrophages stimulated by TNF-α (100 ng/mL) for the indicated time (Figure 2B). In addition, the supernatants of mouse peritoneal macrophages were collected to perform ELISA analysis after they were pretreated with DMSO or Kumujan B for 3 h followed by TNF-α (100 ng/mL) stimulation. Consistently, pro-inflammatory factor IL-6 also decreased significantly in a dose-dependent manner (Figure 2C) and at different time courses (Figure 2D). The results above indicate that Kumujan B inhibits the production of pro-inflammatory cytokines IL-1β and IL-6 with stimulation by TNF-α.

### 3.3 Kumujan B suppresses TNF-α-induced phosphorylation of JNK in mouse peritoneal macrophages

**FIGURE 3:**
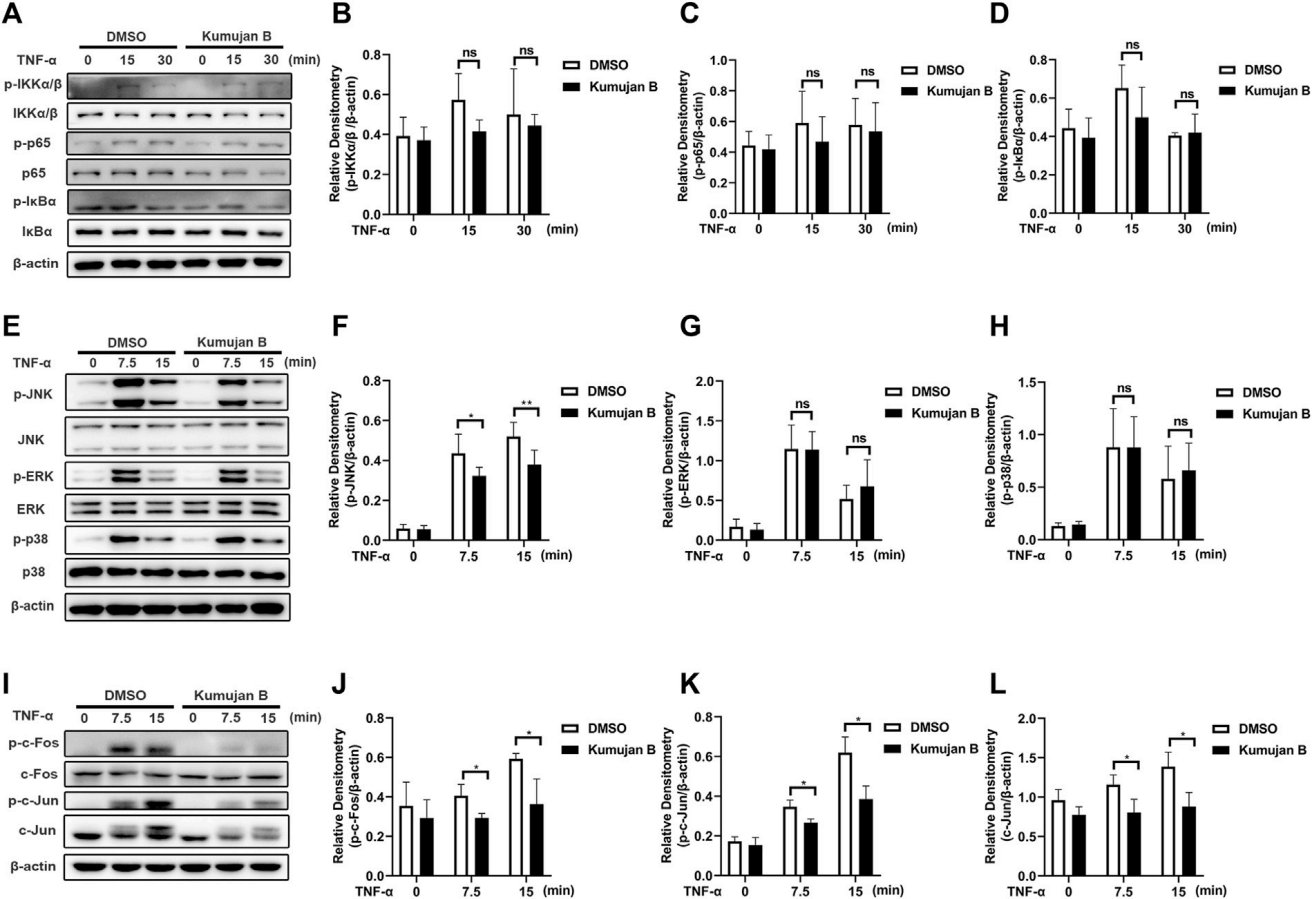
Kumujan B suppresses TNF-α-induced inflammatory response via phosphorylation of JNK in mouse peritoneal macrophages. **(A–D)** Mouse peritoneal macrophages were pretreated with DMSO or Kumujan B (25 μM) for 3 h, followed by stimulation by TNF-α (100 ng/mL) for 0 min, 15 min, or 30 min. Western blotting was used to determine the expression of indicated proteins. **(E–L)** Mouse peritoneal macrophages were pretreated with DMSO or Kumujan B (25 μM) for 3 h followed by stimulation by TNF-α (100 ng/ml) for 0 min, 7.5 min, or 15 min. Western blot assay and statistical analysis were used to detect the expression of indicated proteins. All results obtained from three independent experiments, and values are presented as the mean ± SD. *p < 0.05, **p < 0.01.

In TNF-α-induced inflammation, NF-κB and MAPK signaling pathways are two major regulators initiating the production of transcription factors and pro-inflammatory cytokines (Zelová and Hošek, 2013). In order to further elucidate the underlying mechanism of how Kumujan B plays a role in inhibiting the expression of inflammatory factors, we first investigated the effect of Kumujan B on the NF-κB signaling pathway. Mouse peritoneal macrophages were pretreated with DMSO or Kumujan B (25 μM) for 3 h, followed by stimulation with TNF-α (100 ng/ml) for 0 min, 15 min, and 30 min. As shown in Figures 3A–D, Kumujan B had little inhibitory effect on the phosphorylation of IKKα/β, p65, and IκBα. Thus, Kumujan B did not affect the activation of the NF-κB signaling pathway. The activation of the MAPK signaling pathway stimulated by TNF-α can also cause the production of pro-inflammatory cytokines IL-1β and IL-6. Western blot assay was used to determine the quantities of JNK, ERK, p38, and their phosphorylation formations on the protein level. We pretreated mouse peritoneal macrophages with DMSO or Kumujan B (25 μM) for 3 h and then stimulated them with TNF-α (100 ng/mL) for 0 min, 7.5 min, and 15 min. Kumujan B significantly suppressed the phosphorylation of JNK in the MAPK signaling pathway (Figures 3E–H). The phosphorylation of JNK led to the activation of transcription factor activator protein-1 (AP-1), which is majorly constituted of c-Fos and c-Jun that both regulate the transcription of pro-inflammatory cytokines (Cui et al., 2007; Ye et al., 2014; Tewari et al., 2018). Therefore, we detected the total c-Fos, c-Jun, and phosphorylation of c-Fos and c-Jun with Western blot assays. It was shown that Kumujan B inhibited TNF-α-induced phosphorylation of c-Fos, c-Jun, and total c-Jun in mouse peritoneal macrophages (Figures 3I–L). These results suggest that Kumujan B suppresses the inflammation induced by TNF-α by inhibiting the expression of the phosphorylation of JNK.

### 3.4 Kumujan B promotes c-Jun degradation in mouse peritoneal macrophages

**FIGURE 4:**
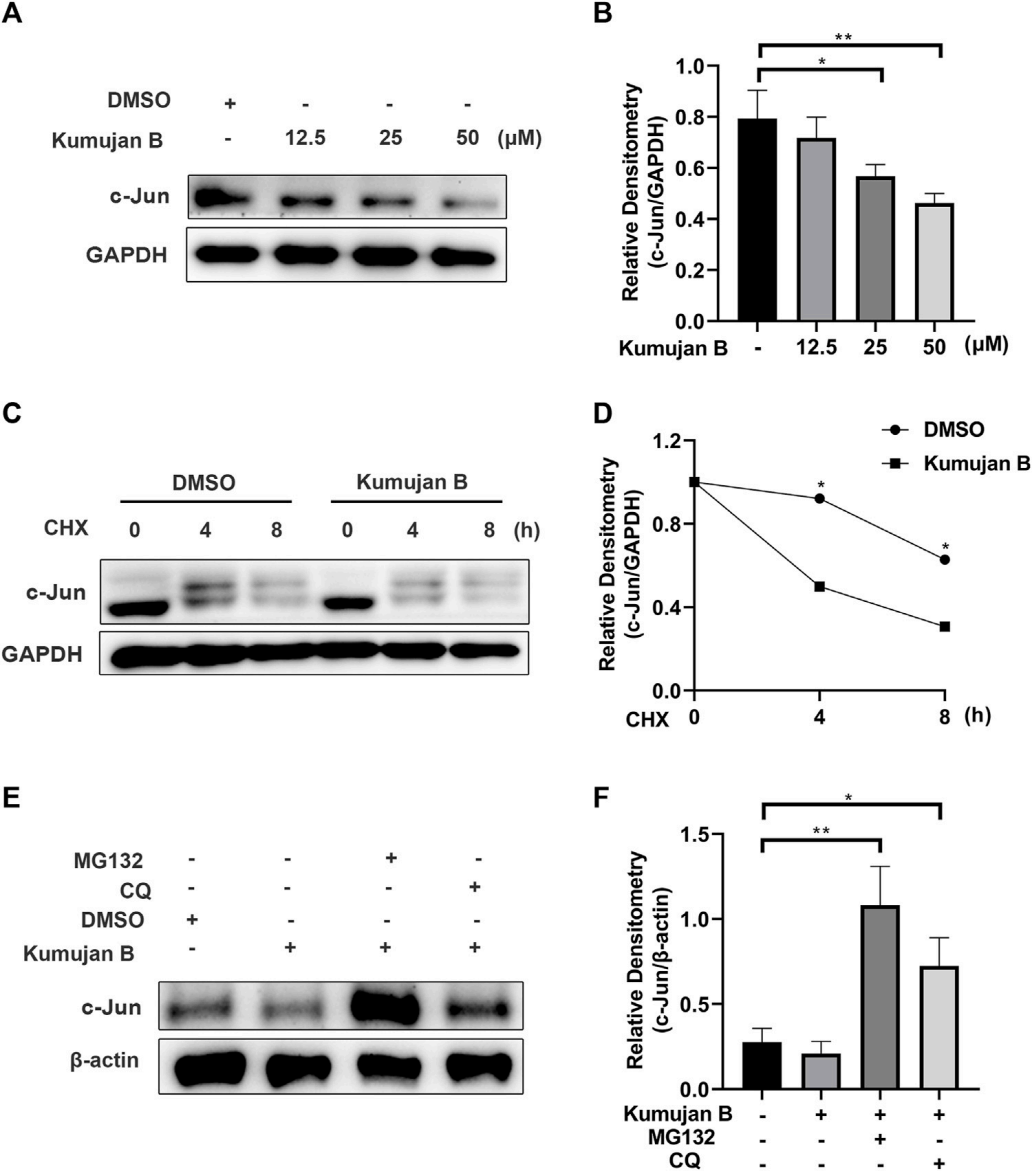
Kumujan B promotes c-Jun degradation. **(A)** Mouse peritoneal macrophages were treated with DMSO or Kumujan B (12.5 μM, 25 μM, or 50 μM) for 4 h and then analyzed by Western blotting. **(B)** The statistical analysis result of Western blotting. **(C, D)** Mouse peritoneal macrophages were pretreated with DMSO or Kumujan B (25 μM) for 3 h and then treated with CHX (100 ng/ml) for the indicated time. Western blotting was used to measure the expression of c-Jun. **(E, F)** Mouse peritoneal macrophages were pretreated with DMSO or Kumujan B (25 μM) for 3 h and then treated with MG132 (5 μM) or CQ (12.5 μM) for 4 h. Immunoblot analysis of c-Jun expression. All results were obtained from three independent experiments, and values are presented as the mean ± SD. *p < 0.05 and **p < 0.01.

In order to elucidate the effects of Kumujan B on the expression of the protein c-Jun, we treated mouse peritoneal macrophages with different concentrations of Kumujan B (0 μM, 12.5 μM, 25 μM, and 50 μM) for 4 h. Western blot analysis is shown in Figures 4A, B. The results showed that the protein level of c-Jun decreased in a dose-dependent manner. Next, we explored whether Kumujan B had effects on the synthesis or degradation of c-Jun. Mouse peritoneal macrophages were treated with cycloheximide (CHX, an inhibitor of protein synthesis) for the indicated time (Di et al., 2021). The half-life of c-Jun was shortened upon treatment with Kumujan B, which blocked its synthesis pathway (Figures 4C, D). These results indicated that Kumujan B could promote the degradation of c-Jun. Generally, there are two protein degradation pathways: proteasomal and lysosomal (Zhao et al., 2022). Mouse peritoneal macrophages were treated with proteasome inhibitor MG132 and autophagy inhibitor CQ. The results showed that Kumujan B promoted the degradation of c-Jun in the ubiquitin-proteasome-mediated pathway, as only the addition of MG132 rescued the expression of c-Jun (Figures 4E, F).

### 3.5 Kumujan B interacts with c-Jun and promotes K11-linked ubiquitination of c-Jun

**FIGURE 5:**
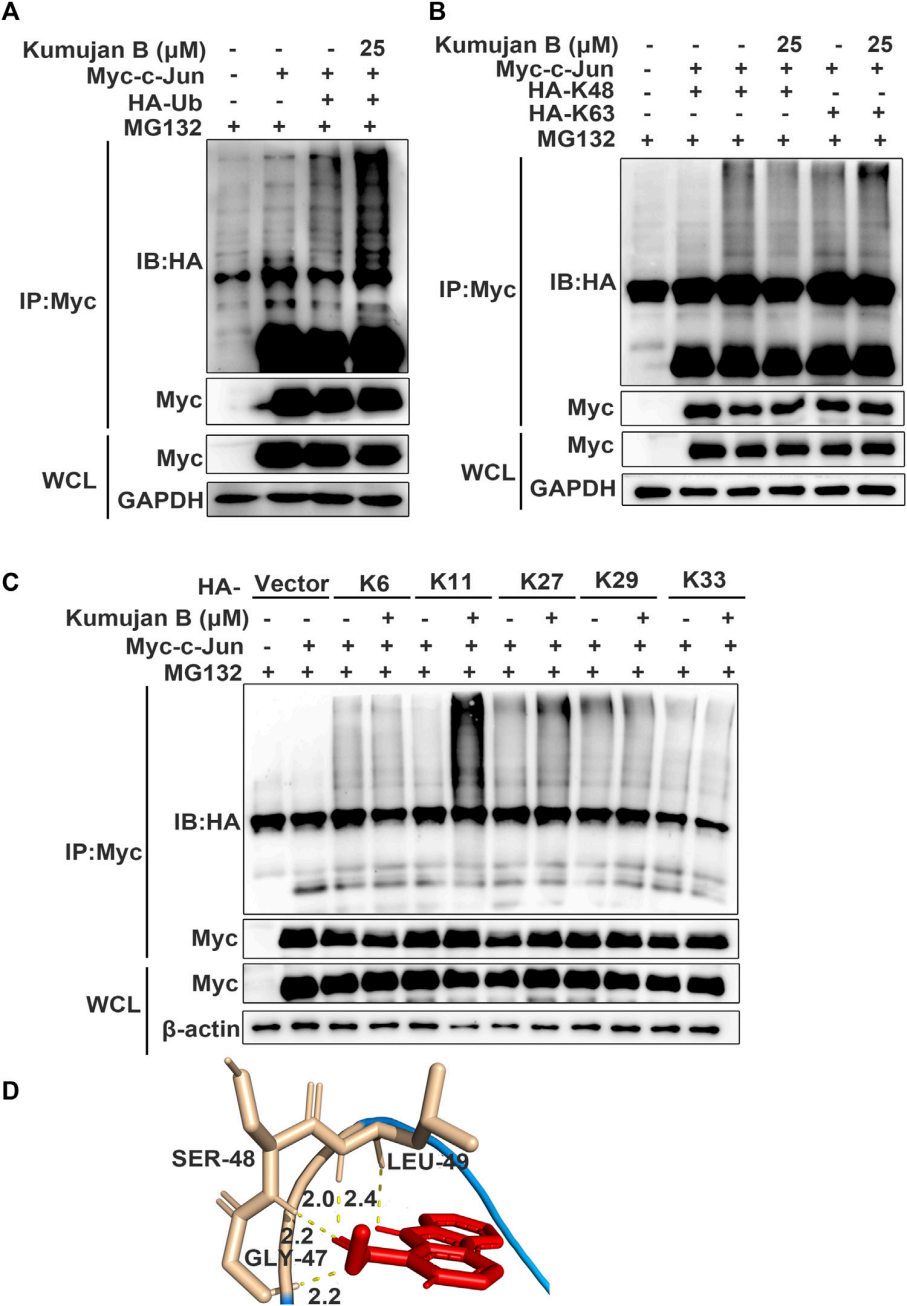
Kumujan B promotes c-Jun degradation via K11-linked ubiquitination. **(A–C)** HEK293T cells were transfected with HA-tagged ubiquitin (HA-Ub) **(A)** or HA-tagged K48 (HA-K48) and HA-tagged K63 (HA-K63) **(B),** or HA-tagged K6 (HA-K6), HA-tagged K11 (HA-K11), HA-tagged K27 (HA-K27), HA-tagged K29 (HA-K29), and HA-tagged K33 (HA-K33) **(C)** with or without Myc-c-Jun for 24 h. Cells were pretreated with MG132 (40 μM) for 1 h, followed by DMSO or Kumujan B (25 μM) for 4 h. Cells were harvested by IP with anti-Myc antibody and protein A/G beads and then probed with anti-HA. **(D)** Prediction binding mode of Kumujan B and c-Jun. All results were obtained from three independent experiments, and values are presented as the mean ± SD.

A previous study shows that post-translational ubiquitin (Ub) is a mark of protein degradation (Popovic et al., 2014). Ubiquitination also plays a crucial role in TNF-mediated inflammatory signaling and cell death regulation (Cockram et al., 2021). HEK293T cells were pretreated with MG132 for 1 h and then co-transfected with HA-Ub plasmids and Myc-c-Jun plasmids following the treatment with DMSO or Kumujan B. Co-immunoprecipitation analysis illustrated that Kumujan B markedly increased the ubiquitination of c-Jun (Figure 5A). Seven Lys residues of Ub can be ubiquitinated, giving rise to isopeptide-linked ubiquitin chains, including K6, K11, K27, K29, K33, K48, and K63. K48 and K63 are major ubiquitin chains in cells (Grice and Nathan, 2016). However, Kumujan B had little influence on the formation of K48-linked ubiquitination and the K63-linked ubiquitination (Figure 5B). Therefore, we analyzed the remaining Lys residues and found that Kumujan B significantly increased the K11-linked ubiquitination of c-Jun in HEK293T cells (Figure 5C). K11-linked polyubiquitin chains are the third most abundant linkages in cells and are relevant to signaling transduction, protein stability, trafficking, and subcellular distribution (Grice and Nathan, 2016; Swatek and Komander, 2016; Deng et al., 2022). We then used molecular docking software to predict the potential interactions between Kumujan B and c-Jun. Kumujan B formed hydrogen bonds with the GLY-47, SER-48, and LEU-49 residues of c-Jun (Figure 5D). In summary, Kumujan B promotes the degradation of c-Jun mainly via the proteasome way through K11-linked ubiquitination. c-Jun residues GLY-47, SER-48, and LEU-49 bind with Kumujan B.

### 3.6 Kumujan B relieves the severity of DSS-induced colitis in mice

**FIGURE 6:**
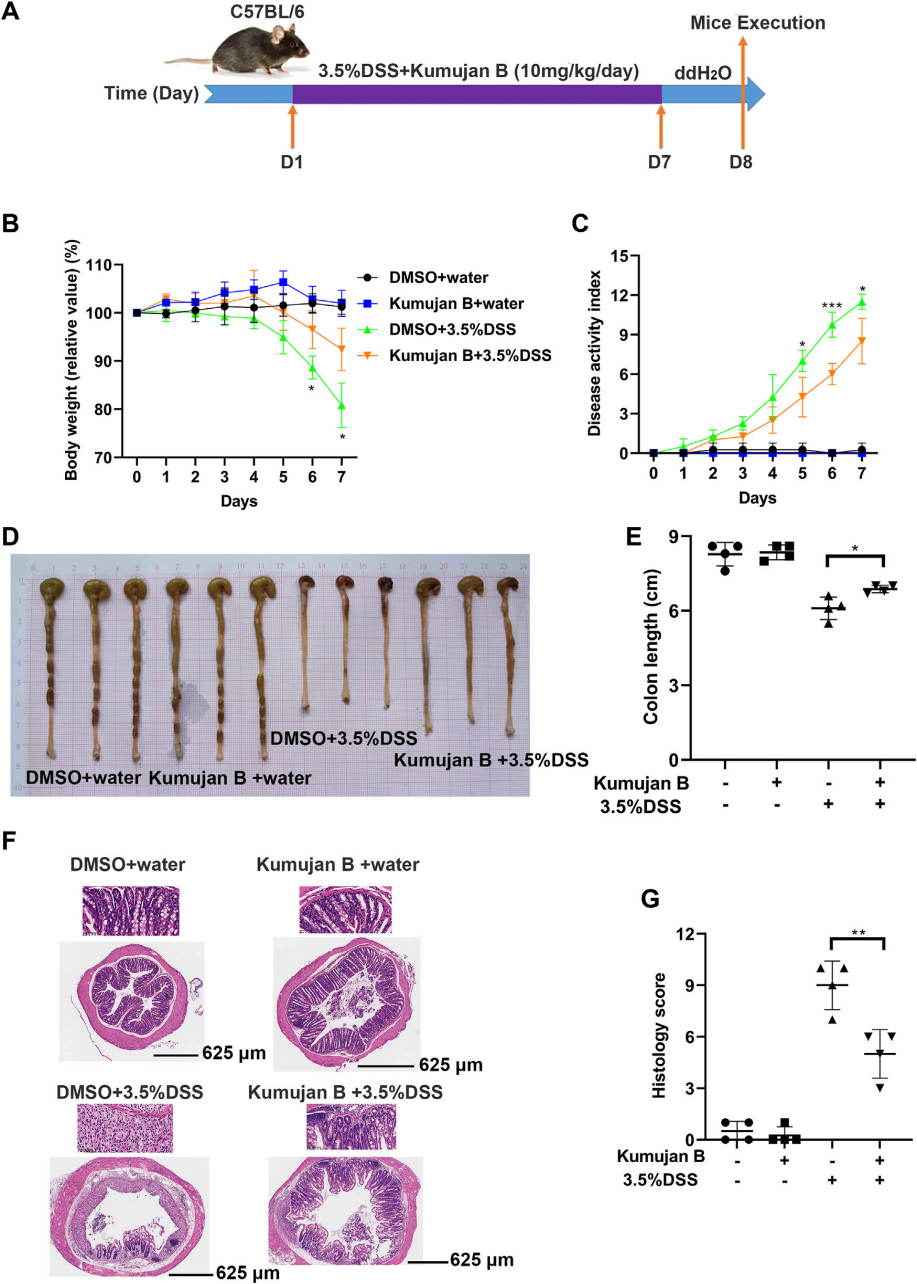
Kumujan B relieves the severity of DSS-induced experimental mice colitis. **(A)** C57BL/6 female mice (n = 4 per group) were fed with distilled water or 3.5% DSS dissolved in distilled water and were intragastric injected with DMSO or Kumujan B (10 mg/kg) per day. After 7 days, all groups were given distilled water for 1 day. On the eighth day, mice were sacrificed, and colons were collected. **(B)** The body weight of each group. **(C)** The DAI score, summarizing stool consistency, gross rectal bleeding, and body weight, was assessed every day. **(D, E)** The representative colons of each group at the time of the necropsy. Their length was measured **(D)** and counted **(E)**. **(F, G)** Representative images of H&E staining of colon sections (bottom: 4×, upper: 40×) **(F)** and lesions **(G)**. Values are presented as mean ± SD. *p < 0.05, **p < 0.01, and ***p < 0.001.

Next, we explored the *in vivo* effect of Kumujan B. We injected DMSO or Kumujan B (10 mg/kg/day) intragastrically to assess the *in vivo* toxicity of Kumujan B. We detected the serum markers of heart (CK) (Supplementary Figure S1A), liver (AST) (Supplementary Figure S1B) and kidney (CR) (Supplementary Figure S1C). No significant difference was found between the DMSO group and the Kumujan B group. H&E staining also showed no significant change in the mice hearts, livers, spleens, lungs, or kidneys (Supplementary Figure S1D) in the Kumujan B-treated group. All of these prove that Kumujan B has no toxic effect *in vivo*. To explore the potential anti-inflammatory response of Kumujan B on mice with induced colitis, we supplied 3.5% DSS freely to the DMSO +3.5% DSS group and the Kumujan B (10 mg/kg/day) +3.5% DSS group (Figure 6A). This animal model is similar to clinical colitis in both symptoms and disease pathology. It can be used to evaluate the efficiency of the therapy method (Kim et al., 2012). As illustrated in Figure 6B, the body weight of the DMSO +3.5% DSS group decreased more rapidly than that of the Kumujan B+ 3.5% DSS group. Compared with water-treated groups, the disease activity index (DAI) score of the groups treated with 3.5% DSS increased consistently, while DAI scores of the Kumujan B+ 3.5% DSS group increased more slowly (Figure 6C). In addition, the colon length was longer in the Kumujan B-treated group than in the DMSO-treated group compared to the 3.5% DSS-treated groups (Figures 6D, E). Histological examination of colon tissue revealed that the DMSO +3.5% DSS group experienced severe epithelial injury, crypt destruction, inflammatory cell infiltration, and higher clinical scores. However, the Kumujan B-treated group with clinical colitis showed milder symptoms in regard to the features mentioned above. (Figures 6F, G). Collectively, Kumujan B relieves colitis symptoms induced by DSS in mice.

### 3.7 Kumujan B attenuated DSS-induced inflammatory responses and protected the epithelial barrier in mice

**FIGURE 7:**
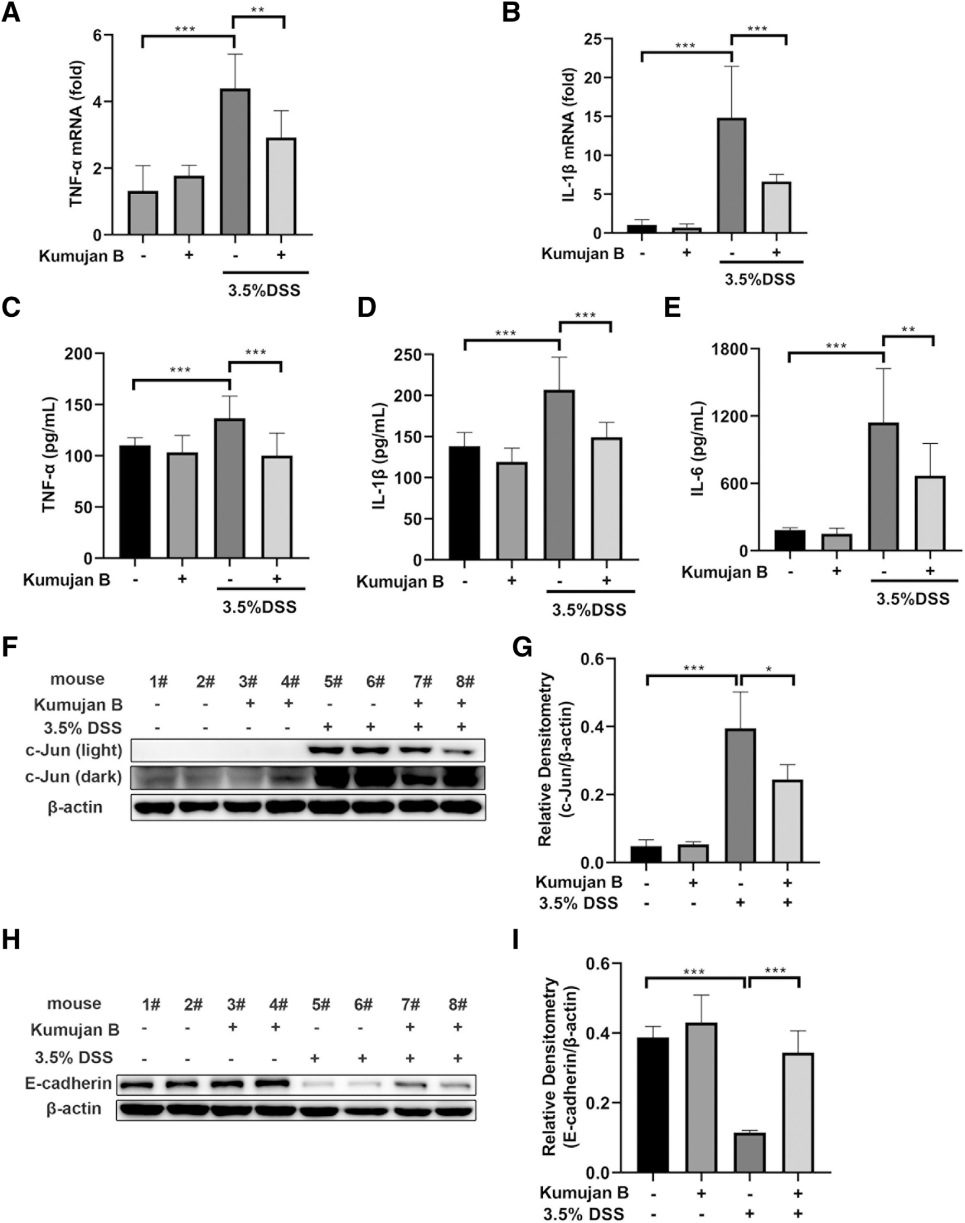
Kumujan B relieves DSS-induced inflammatory responses and protects the epithelial barrier in mice. **(A, B)** Q-PCR was used to analyze the expression of TNF-α **(A)** and IL-1β **(B)** in colon tissue. **(C–E)** ELISA determined the expression of TNF-α **(C)**, IL-1β **(D),** and IL-6 **(E)** in colon tissue. **(F–I)** Western blot assay was used to detect the expression of c-Jun **(F, G)** and E-cadherin **(H, I)** in colon tissues. Values are presented as the mean ± SD. *p < 0.05, **p < 0.01, and ***p < 0.001.

TNF-α, IL-6, and IL-1β are highly expressed in experimental mice colitis (Marafini et al., 2019). To further evaluate the anti-inflammatory effects of Kumujan B on DSS-induced colitis mice, we assessed the expression of pro-inflammatory cytokines in mice colon tissues. DSS triggered the increase of TNF-α and IL-1β in the mRNA level, while gavaging with Kumujan B inhibited this trend. (Figures 7A, B). Likewise, the secretions of TNF-α, IL-1β, and IL-6 increased upon DSS treatment, while Kumujan B retarded these secretions (Figures 7C–E). Furthermore, Kumujan B attenuated the expression of c-Jun, a transcription factor associated with inflammatory response (Figures 7F, G). Tight junctions can maintain the intestinal barrier. As Figures 7H, I show, protein expression of E-cadherin significantly decreased in colon tissues in the DMSO +3.5% DSS group, while Kumujan B treatment reversed this trend. These results suggest that Kumujan B alleviates DSS-induced inflammatory responses and protects the epithelial barrier.

## 4 Discussion

UC and CD are two of the most important entities in IBD. In animal models, acute IBD can be modeled by feeding DSS polymers in drinking water, which induces weight loss, diarrhea, bloody feces, and shortening of colon length, which is similar to human IBD. In our study, Kumujan B alleviated mice weight loss, DAI score, and shortening of colon length, effectively delaying the progress of the disease (Bauer et al., 2010; Mizoguchi, 2012).

The intestinal epithelium forms a solid physical barrier and maintains intestinal homeostasis (Maloy and Powrie, 2011). Tight junctions play an important role in constituting the barrier function, and epithelial cells, such as E-cadherin, an adherens junction protein, help in maintaining strong cell–cell adhesion (Oshima and Miwa, 2016). In the IBD model, intestinal epithelial injury is a characteristic symptom consisting of mucosal erosion, ulceration, cryptitis, and crypt abscess formation (Garcia-Carbonell et al., 2018). In response to DSS administration, the expression of E-cadherin decreased markedly. Furthermore, the intestinal epithelium displayed severe dysfunction. In contrast to the untreated group, Kumujan B decreased the histological score of colitis-like pathology and increased the expression of E-cadherin in mice colonic tissues, protecting the barrier function in the intestinal epithelium effectively.

When the barrier integrity is destroyed, a series of inflammatory processes are subsequently triggered (Huang et al., 2020). Intestinal epithelial cells (IECs) are exposed to numerous pro-inflammatory cytokines in IBD. Macrophages as antigen-presenting cells (APCs) can be found in inflamed mucosa. Pro-inflammatory cytokines such as IL-1β, IL-6, and TNF-α can be highly produced in macrophages, resulting in a stronger inflammatory condition (Neurath, 2014). Anti-TNF agents as potential emerging biological drugs have broad prospects in curing IBD (Kotla and Rochev, 2023). As shown with *in vitro* studies, Kumujan B suppressed TNF-α-induced inflammation in mouse peritoneal macrophages by inhibiting the expression of IL-1β at the mRNA level and the secretion of IL-6 at the protein level. In animal studies, the expression of IL-1β and TNF-α could be suppressed at both mRNA and protein levels in colon tissues. Meanwhile, the expression of IL-6 could be suppressed at the protein level in colon tissues.

The JNK signaling pathway via AP-1 activation regulates the expression of inflammatory mediators, including TNF-α, which has a close relationship with IBD (Salh, 2007). As a major target of JNK, AP-1 is composed of Fos and Jun family members. In the JNK/Jun pathway, a series of target genes contain AP-1-binding sites (Wagner and Nebreda, 2009). c-Jun is the most potent member in AP-1. c-Jun can be phosphorylated effectively by JNK. Phosphorylated c-Jun triggers the activation of AP-1 (Karin et al., 1997; Meng and Xia, 2011). Kumujan B suppressed the expression of c-Jun in both *in vivo* and *in vitro* research. In mouse peritoneal macrophages, Kumujan B promoted c-Jun degradation via the proteasomal pathway. In a further study, we found that Kumujan B promoted the degradation of c-Jun via K11-linked ubiquitination. We also predicted that the potential binding sites between Kumujan B and c-Jun were hydrogen bonds at GLY-47, SER-48, and LEU-49 residues.

Overall, the present study revealed that Kumujan B, a derivative of β-carboline, exerted protective effects in experimental mice colitis, including suppressing the expression of pro-inflammatory cytokines and increasing the expression of tight junctions. In mouse peritoneal macrophages, Kumujan B inhibited TNF-α-induced inflammation response by regulating the JNK signaling pathway, especially by promoting the degradation of c-Jun by K11-linked ubiquitination. Consequently, pro-inflammatory cytokines IL-1β and IL-6 were downregulated upon Kumujan B treatment. These findings suggest that Kumujan B can be a potential competent anti-inflammatory candidate for IBD therapy.

## Figures and Tables

**Table:** 

**IBD**	inflammatory bowel disease
**CD**	Crohn’s disease
**UC**	ulcerative colitis
**5-ASA**	5-aminosalicylic acid
**TNF-α**	tumor necrosis factor α
**TNFR1**	TNF receptor 1
**TNFR2**	TNF receptor 2
**TRADD**	TNF receptor-associated death domain
**RIP**	receptor-interacting protein
**IκBα**	inhibitor of NF-κB
**MAPKKK/MEKK**	mitogen-activated protein kinase kinase kinases
**MAPKK/MEK**	MAPK kinases
**MAPK**	mitogen-activated protein kinases
**JNK**	c-Jun N-terminal protein kinases
**ERK**	extracellular regulated protein kinases
**AP-1**	activator protein 1
**IL-1β**	interleukin 1β
**IL-6**	interleukin 6
**WHO**	World Health Organization
**DMSO**	methyl sulfoxide
**CHX**	cycloheximide
**MG132**	Z-Leu-D-Leu-Leu-al
**CQ**	chloroquine
**DSS**	dextran sulfate sodium salt
**DAI**	disease activity index
**CCK8**	cell counting kit-8
**IECs**	intestinal epithelial cells
**APCs**	antigen-presenting cells
**AST**	glutamic oxaloacetic transaminase
**CK**	creatine kinase
**CR**	creatinine

## Data Availability

The raw data supporting the conclusions of this article will be made available by the authors, without undue reservation.
