# Supplementary material for: Kumujan B suppresses TNF-α-induced inflammatory response and alleviates experimental colitis in mice
Source: Front Pharmacol. 2024 Aug 1;15:1427340. doi: 10.3389/fphar.2024.1427340 (PMC11324439; doi:10.3389/fphar.2024.1427340)
Supplement: Supplementary file 1 [file DataSheet1.docx]

**Supplementary material**

**Figure S1**


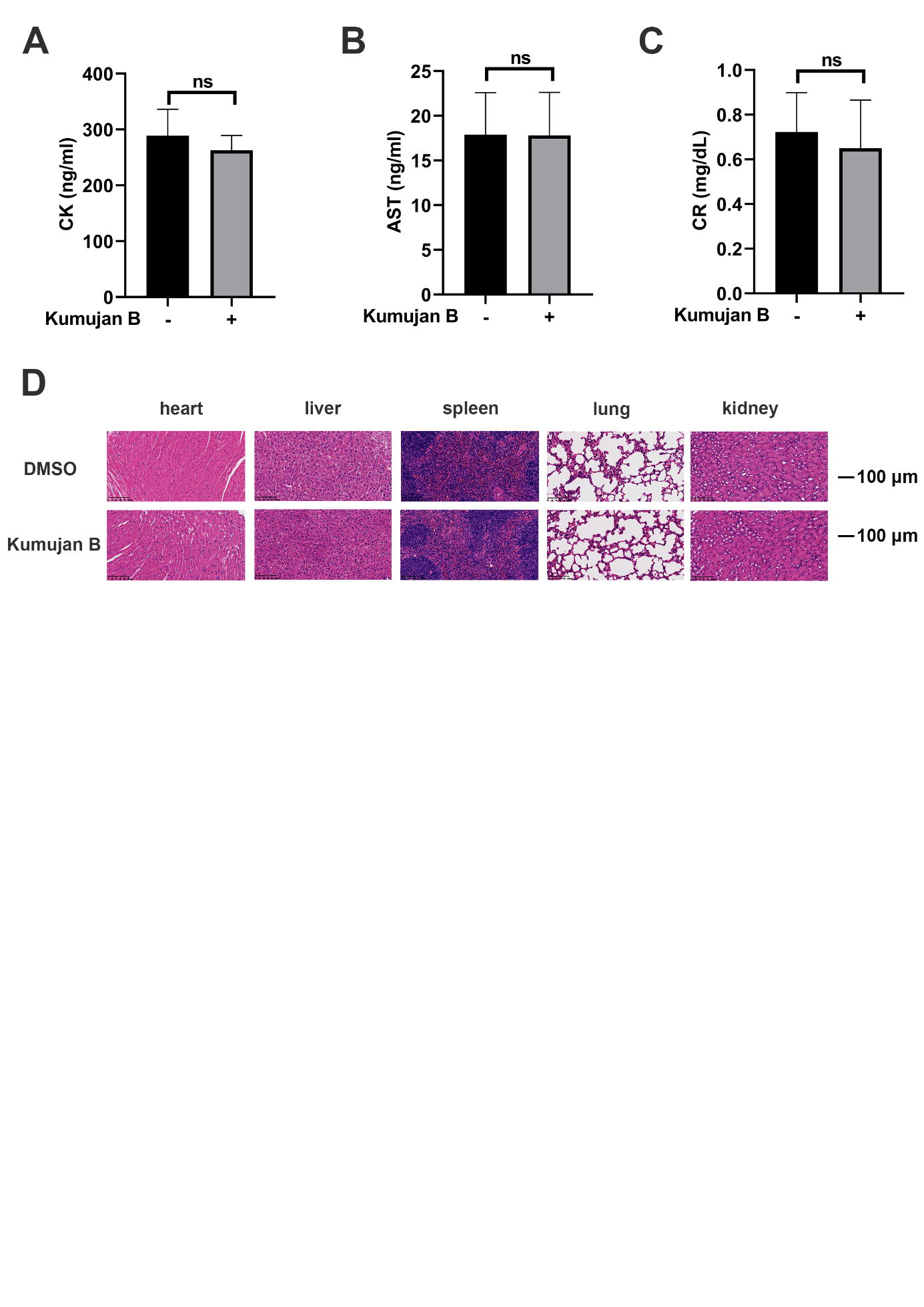


**Supplementary Figure S1. In vivo toxicity detection of Kumujan B in mice.** (A-C) C57BL/6 female mice (n=4 per group) were intragastric injected with DMSO or Kumujan B (10 mg/kg/day). The mice serums were collected and were used to detect the expression of CK (A), AST (B) and CR (C). (D) Representative images of H&E staining of mice heart, liver, spleen, lung and kidney (20x). Values were presented as the mean ± SD. ns, not significant.
